# Causal association between body mass index and risk of colon polyps: A Mendelian randomization study

**DOI:** 10.1097/MD.0000000000042022

**Published:** 2025-05-23

**Authors:** Xin-Jie Ning, Jian-Feng Li, Hui-Long Guo, Jing-Yao Chen

**Affiliations:** a The Seventh Affiliated Hospital of Sun Yat-sen University, Shenzhen, China; b Digestive Diseases Center, The Seventh Affiliated Hospital, Sun Yat-sen University, Shenzhen, China; c Emergency and Disaster Medicine Center, The Seventh Affiliated Hospital of Sun Yat-sen University, Shenzhen, Guangdong Province, China.

**Keywords:** body mass index, colon polyps, colorectal cancer, Mendelian randomization

## Abstract

The relationship between obesity and colorectal polyps remains inconclusive. This study aimed to examine whether body mass index (BMI) is causally associated with colon polyps. A two‐sample Mendelian randomization (MR) analysis using the inverse variance weighted, weighted median and MR‐Egger regression methods was performed. We used the publicly available summary statistics data sets of genome-wide association studies (GWAS) meta‐analyses for BMI in individuals of European descent (n = 322,154; GIANT consortium) as the exposure and a GWAS for colon polyps included in the UK Biobank (total n = 463,010; case = 4779, control = 458,231) as the outcome. We selected 76 single nucleotide polymorphisms at genome‐wide significance from GWASs on BMI as the instrumental variables. The inverse variance weighted method showed evidence to support a causal association between BMI and colon polyps (beta = 0.002, SE = 0.001, *P* = .012). MR‐Egger regression revealed that directional pleiotropy was unlikely to be biasing the result (intercept = ‐9.3e‐06;*P* = .889), but it showed no causal association between BMI and colon polyps (beta = 0.002, SE = 0.002, *P* = .09). However, the weighted median approach yielded evidence of a causal association between BMI and colon polyps (beta = 0.001, SE = 0.001, *P* = .01). Cochran Q test and the funnel plot indicated no evidence of heterogeneity and asymmetry, indicating no directional pleiotropy. The results of MR analysis support that BMI may be causally associated with an increased risk of colon polyps.

## 1. Introduction

Colorectal cancer (CRC) is the third most common cause of cancer mortality worldwide with more than 1.85 million cases and 850,000 deaths annually.^[1]^ Based on Morson adenoma-carcinoma hypothesis, colon polyps are presently regarded as the primary etiological factor in the development of colorectal cancer.^[2]^ Hence, comprehending the risk factors and pathogenesis associated with colon polyps holds paramount importance in mitigating the incidence of colon polyps and potentially colorectal cancer.

The pathogenesis of colon polyps is currently unclear, although it is known to be associated with various risk factors such as chronic inflammatory stimuli, genetic factors, metabolic syndrome, long-term constipation, and colon melanosis. The relationship between obesity and colorectal polyps has been a topic of extensive research. However, the findings regarding this relationship remain inconclusive. A study conducted in 2018 revealed that asymptomatic patients with a body mass index (BMI) of 25 to 30 kg/m^2^ had a higher likelihood of developing colorectal adenomatous polyps compared to patients with a lower BMI.^[3]^ However, according to a 2017 study conducted in the United States, there was no significant change in the occurrence of colorectal polyps with an increase in BMI.^[4]^ A Korean article reported negative results regarding the relationship between BMI, hypertriglyceridemia, hypercholesterolemia, and the development of colorectal polyps.^[5]^ These findings suggest that there are contradictions in the connection between obesity and colon polyps, highlighting the need for further research in this area.

Mendelian randomization analysis aims to assess the causal impact of exposure on outcomes by utilizing genetic variation as instrumental variables (IVs).^[6]^ Typically, single nucleotide polymorphisms (SNPs) are the commonly employed genetic variants for this purpose. Randomized controlled trials (RCTs) are considered the gold standard for clinical evidence. In a similar manner, Mendelian randomization (MR) mimics RCTs, with SNPs playing a role similar to randomization in an RCT. Individuals with different SNPs are randomly assigned to subgroups. Since SNPs are formed during embryonic development and are less influenced by acquired factors, they can help balance the confounding factors between subgroups, resulting in an effect that is comparable to random grouping.

This study aims to investigate the causal relationship between BMI and colon polyps using genome-wide and epigenome-wide association study (GWAS/EWAS) data. The MR method will be employed, and BMI-related genetic variants will be used as instrumental variable.

## 2. Material and methods

### 2.1. Study design

To evaluate the association between BMI and colon polyps, we performed a two-sample MR study. Instrumental variables of BMI and colon polyps were derived from large-scale GWASs. Summary-level data on colon polyps were obtained from the UK Biobank, while that for BMI was derived from a large-scale genome-wide association study. The random effects inverse-variance weighted MR approach was applied as the primary method, along with weighted median, MR-Egger, MR-PRESSO methods as sensitivity analyses.

Three main assumptions should be considered when conducting MR analysis: (1) instrumental variables are strongly correlated with exposures of interest; (2) instruments are not related to the potential confounders; (3) the selected genetic variants should affect the outcome only via the exposures of interest.

### 2.2. Patient and public involvement

Currently, patient and public are not suitable to participate in this project.

### 2.3. Exposure and outcome data source

We used the publicly available summary statistics data sets of GWAS meta-analyses for BMI in individuals of European descent (n = 339,224; GIANT consortium) as the exposure.^[7]^ We obtained summary statistics (beta coefficients and standard errors) for 76 SNPs associated with BMI as the IVs from the GWASs on BMI. We used the publicly available summary statistic data sets of a GWAS for colon polyps included in the UK Biobank (total n = 463,010; case n = 4779, control n = 458,231) (Dataset: ukb-b-1968) as the outcome.

### 2.4. Genetic variants selection criteria

SNPs associated with BMI at the genome-wide significance threshold (*P* < 5 * 10^‐8^) were identified, linkage disequilibrium (*r*^2^ > 0.01 or clump distance < 10,000 kb). We calculated F-statistics for each SNP to exclude weak instrumental variables (F < 10), and no weak instrumental variable was found. Thus, weak instrument bias was negligible.

### 2.5. Statistical analysis

The random effect inverse-variance weighted MR method was used as the primary method, and MR estimates were performed in beta values because the exposure and outcome are all binary variables.^[8]^ The MR analysis was done using the MR-Base platform.^[9]^ The conventional random effects inverse variance weighted (IVW) analysis was used for the MR analysis. Heterogeneity of the causal estimates across all SNPs was tested by Q statistics. The effect of each SNP is presented in a forest plot. MR Egger analysis is also shown in the forest plots, which suffers from low statistical power. The effect of each single instrument on the overall effect was shown in the plot of the leave-one-out analysis. Several sensitivity analyses, including the weighted median,^[10]^ MR-Egger,^[11]^ and MR-PRESSO^[12]^ methods, were conducted to examine the consistency of results and to detect horizontal pleiotropy. The weighted median method can provide consistent causal estimates if more than half of the weight comes from valid instruments. MR Egger regression can detect horizontal pleiotropy by its intercept and generate an estimate with adjustment for pleiotropic effects; however, it has less statistical power. The MR-PRESSO method can detect SNPs that are outliers and provide a causal estimate after the removal of these outliers. The embedded distortion test can detect the difference between estimates before and after the removal of outliers. Besides, Cochrane Q test was used to assess the heterogeneity among estimates of SNPs in one analysis.

## 3. Results

### 3.1. Instrumental variables for Mendelian randomization

We obtained the relevant information of 76 SNPs significantly related to BMI (see Tables 1 and 2 for original data): SNP, effective allele (EA), non-EA, effective allele frequency, effect size of allele (beta, β），*P*-value. The total *R*^2^ of the original 76 SNPs was 1.09%, all SNPs were strong Instrumental variables estimation (F > 10), and the total F value of SNPs was 5049.62 (Table S1, Supplemental Digital Content, https://links.lww.com/MD/O996).

**Table 1:** Information of 76 SNPs significantly related to BMI.

SNP	EAF	beta	SE	*R* ^2^	F
rs1000940	0.225	0.0184	0.0033	6.71411E-05	31.09
rs10132280	0.3333	-0.0221	0.0033	9.68555E-05	44.85
rs1016287	0.675	-0.0228	0.0033	0.000103088	47.74
rs10182181	0.5	0.0309	0.0029	0.000245146	113.53
rs10733682	0.575	-0.0188	0.003	8.48098E-05	39.27
rs10840100	0.725	0.0206	0.003	0.000101826	47.15
rs11030104	0.2	-0.0416	0.0037	0.000272944	126.41
rs11057405	0.0917	-0.0304	0.0053	7.10517E-05	32.90
rs11165643	0.575	0.0221	0.003	0.000117193	54.27
rs11672660	0.175	-0.0339	0.0038	0.000171857	79.58
rs1167827	0.5417	0.02	0.0031	8.98891E-05	41.62
rs11727676	0.075	-0.0365	0.0063	7.24908E-05	33.57
rs12286929	0.4333	0.0211	0.0029	0.000114322	52.94
rs12429545	0.1	0.0324	0.0044	0.000117096	54.22
rs12448257	0.225	0.0246	0.0037	9.5463E-05	44.20
rs12940622	0.4583	-0.0183	0.0029	8.5996E-05	39.82
rs12986742	0.5	0.0207	0.0036	7.14026E-05	33.06
rs13021737	0.875	0.0604	0.0039	0.000517761	239.85
rs13078960	0.1833	0.029	0.0038	0.000125772	58.24
rs13107325	0.1167	0.0472	0.0066	0.000110448	51.14
rs13130484	0.4333	0.0398	0.003	0.000379987	176.00
rs13191362	0.2	-0.0285	0.0047	7.94089E-05	36.77
rs13201877	0.0833	0.0236	0.0043	6.50532E-05	30.12
rs13329567	0.2167	-0.0307	0.0035	0.000166142	76.94
rs1421085	0.45	0.0803	0.003	0.001544994	716.45
rs1441264	0.55	0.0172	0.0031	6.64836E-05	30.78
rs1460676	0.2167	0.0209	0.0038	6.53291E-05	30.25
rs14810	0.675	0.0183	0.0033	6.64133E-05	30.75
rs1516725	0.9083	0.0448	0.0044	0.000223853	103.67
rs1528435	0.5833	0.0175	0.003	7.34871E-05	34.03
rs17001654	0.1583	0.0304	0.0052	7.38105E-05	34.18
rs17066856	0.1333	-0.0371	0.005	0.000118896	55.06
rs17094222	0.2083	0.0249	0.0037	9.78053E-05	45.29
rs17203016	0.2	0.0211	0.0038	6.65853E-05	30.83
rs17381664	0.425	0.0201	0.0031	9.07902E-05	42.04
rs17724992	0.3083	-0.0196	0.0034	7.17683E-05	33.23
rs1928295	0.425	-0.0182	0.0029	8.50588E-05	39.39
rs2033529	0.2583	0.0183	0.0032	7.06287E-05	32.70
rs2060604	0.4417	-0.0203	0.003	9.88818E-05	45.79
rs2112347	0.375	-0.0254	0.003	0.000154799	71.68
rs2176598	0.8	-0.0185	0.0033	6.78728E-05	31.43
rs2183825	0.2917	0.0241	0.0032	0.000122487	56.72
rs2365389	0.3417	-0.0195	0.003	9.12424E-05	42.25
rs2820292	0.5083	0.0181	0.0029	8.41268E-05	38.95
rs2836754	0.65	0.0169	0.003	6.85347E-05	31.73
rs2890652	0.125	0.0279	0.0049	7.00157E-05	32.42
rs3736485	0.575	-0.016	0.0029	6.57393E-05	30.44
rs3800229	0.6917	0.0175	0.0032	6.45889E-05	29.91
rs3817334	0.45	0.0256	0.003	0.000157246	72.82
rs3849570	0.3667	0.0183	0.0033	6.64133E-05	30.75
rs3888190	0.3583	0.0311	0.003	0.000232053	107.47
rs4740619	0.4667	-0.017	0.0029	7.42129E-05	34.36
rs4889606	0.3583	-0.0187	0.003	8.391E-05	38.85
rs543874	0.2667	0.0497	0.0037	0.000389538	180.43
rs6091540	0.275	-0.0185	0.0033	6.78728E-05	31.43
rs6457796	0.2583	0.0209	0.0033	8.66237E-05	40.11
rs6477694	0.6417	-0.0169	0.003	6.85347E-05	31.73
rs6567160	0.2833	0.0562	0.0035	0.00055655	257.83
rs657452	0.5833	-0.0227	0.0031	0.000115794	53.62
rs6713510	0.4833	0.0164	0.0029	6.90671E-05	31.98
rs6804842	0.575	0.0183	0.003	8.0359E-05	37.21
rs7138803	0.4417	0.032	0.003	0.000245675	113.78
rs7144011	0.275	0.0274	0.0035	0.000132348	61.29
rs7531118	0.6083	0.0331	0.003	0.000262851	121.73
rs7599312	0.2917	-0.0214	0.0033	9.08176E-05	42.05
rs7715256	0.55	-0.0168	0.0029	7.24771E-05	33.56
rs7903146	0.25	-0.0235	0.0033	0.000109514	50.71
rs879620	0.5917	0.0244	0.0039	8.45324E-05	39.14
rs891389	0.325	0.0209	0.0037	6.89079E-05	31.91
rs9304665	0.7	0.0243	0.0043	6.89692E-05	31.94
rs9374842	0.7417	0.0196	0.0034	7.17683E-05	33.23
rs943005	0.1	0.0444	0.0038	0.000294768	136.52
rs9540493	0.55	-0.0182	0.0031	7.44383E-05	34.47
rs9579083	0.2333	0.0295	0.0046	8.88177E-05	41.13
rs977747	0.5333	-0.0168	0.003	6.77261E-05	31.36
rs9926784	0.2083	-0.0249	0.0038	9.27259E-05	42.94

BMI = body mass index, EAF = effective allele frequency, SNPs = single-nucleotide polymorphisms.

**Table 2:** MR estimates from each method of assessing the causal effect of BMI on the risk of colon ployps.

MR method	Num of SNPs	Beta	Se	pval	OR	Low 95%	Up 95%
MR Egger	76	0.002386	0.002421	0.09048	1.002388849	0.997643621	1.007156647
Weighted median	76	0.001634	0.001688	0.01007	1.001635336	0.998326921	1.004954714
Inverse variance weighted	76	0.002075	0.0009861	0.01217	1.002077154	1.000142254	1.004015798
Weighted mode	76	0.002005	0.001995	0.01942	1.002007011	0.998096614	1.005932729

BMI = body mass index, MR = Mendelian randomization, SNPs = single-nucleotide polymorphisms.

### 3.2. Mendelian randomization results

**Figure 1.**
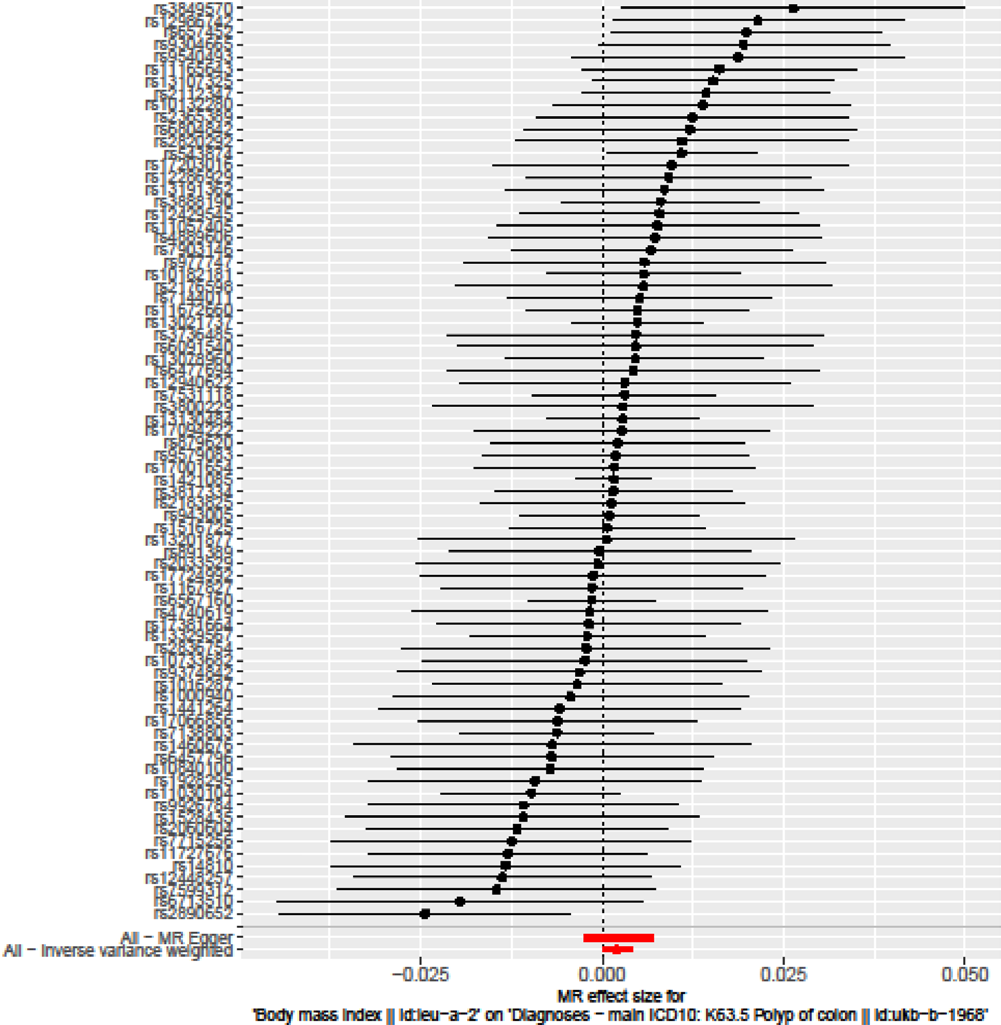
Forest plot of the causal effects of single nucleotide polymorphisms associated with body mass index on colon polyps.

**Figure 2.**
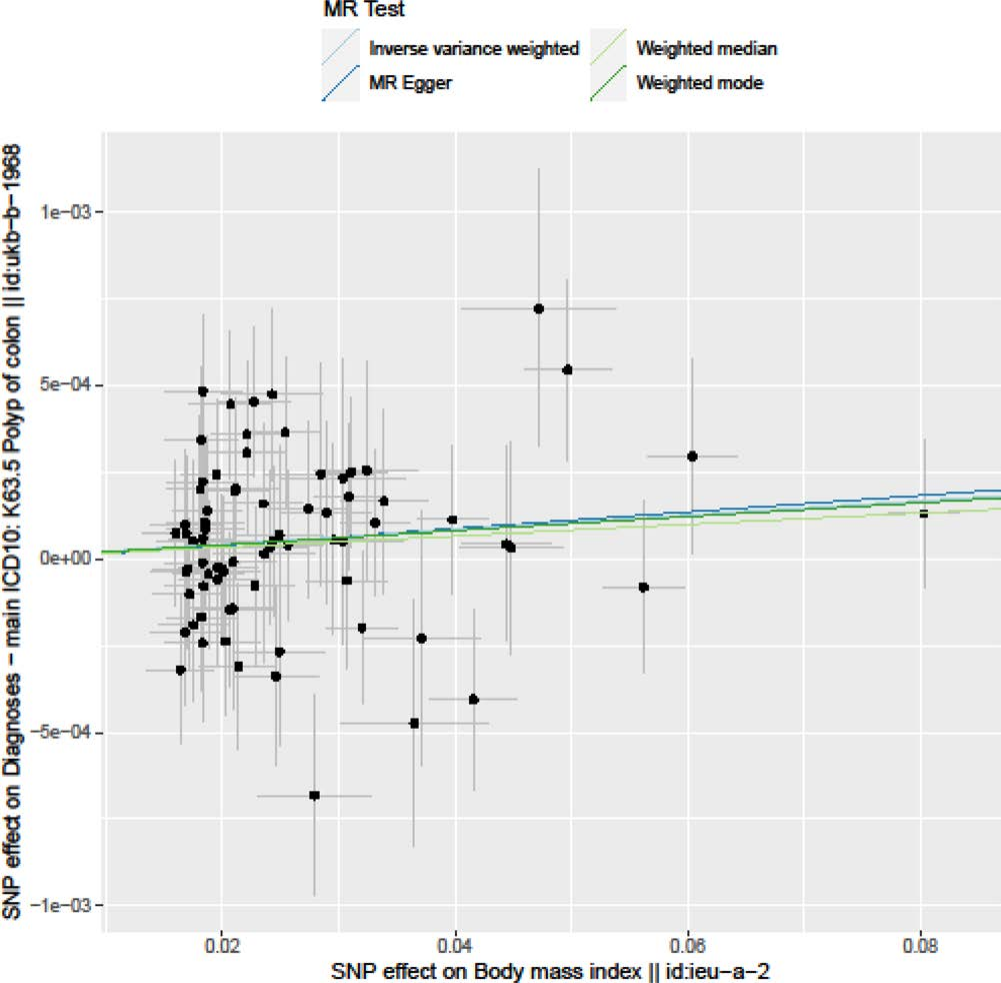
Scatter plots of genetic associations with body mass index against the genetic associations with colon polyps. The slopes of each line represent the causal association for each method. The blue line represents the inverse-variance weighted estimate, the green line represents the weighted median estimate, and the dark blue line represents the Mendelian randomization-Egger estimate.

The IVW method showed evidence to support a causal association between BMI and colon polyps (beta = 0.002, SE = 0.001, *P* = .012; Table 2, Figs. 1 and 2). The intercept represents the average pleiotropic effect across the genetic variants (the average direct effect of a variant with the outcome). An intercept that differs from zero (the MR‐Egger test) is indicative of directional pleiotropy. MR‐Egger regression revealed that directional pleiotropy was unlikely to be biasing the result (intercept = ‐9.3e‐06; se = 6.6e‐05; *P* = .889). The MR‐Egger analysis showed no causal association between BMI and colon polyps (beta = 0.002, SE = 0.002, *P* = .09; Table 2, Figs. 1 and 2). However, the weighted median approach yielded evidence of a causal association between BMI and colon polyps (beta = 0.001, SE = 0.001, *P* = .01; Table 2, Fig. 2). The association between BMI and colon polyps was not consistent between MR Egger and weighted median methods. The IVW and weighted median method suggest a causal effect of BMI on the risk of colon polyps, whereas the MR‐Egger method suggests a null causal effect. Considering that compared to the MR Egger analysis, the weighted median estimator has the advantage of retaining greater precision in the estimates, the results of the MR analysis may support a potential causal association between BMI and colon polyps.

### 3.3. Heterogeneity and sensitivity test

**Figure 3.**
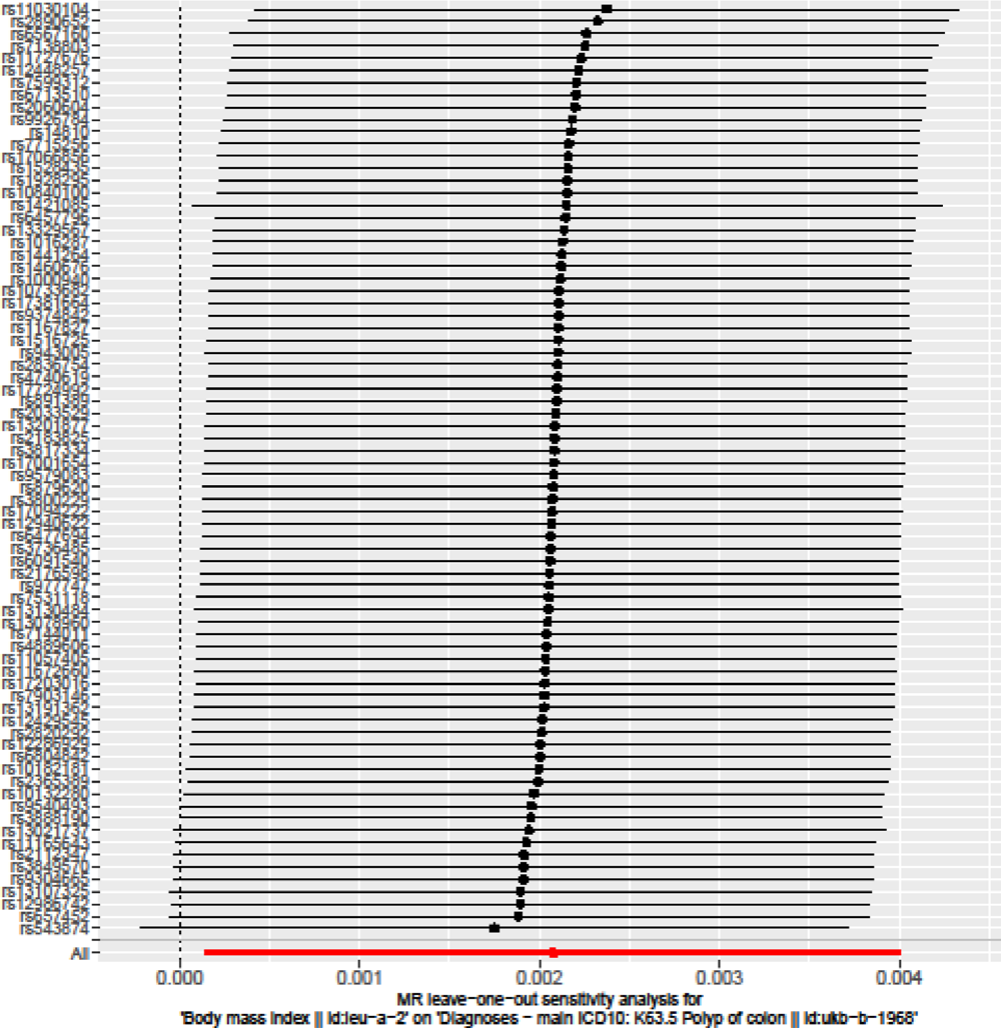
Leave-one-out of SNPs associated with BMI and their risk of colon polyps. Each black point represents result of the IVW MR method applied to estimate the causal effect of BMI on colon polyps excluding particular SNP (rs1000940, rs10132280, rs1016287, rs10182181, rs10733682, rs10840100, rs11030104, rs11057405, rs11165643, rs11672660, rs1167827, rs11727676, rs12286929, rs12429545, rs12448257, rs12940622, rs12986742, rs13021737, rs13078960, rs13107325, rs13130484, rs13191362, rs13201877, rs13329567, rs1421085, rs1441264, rs1460676, rs14810, rs1516725, rs1528435, rs17001654, rs17066856, rs17094222, rs17203016, rs17381664, rs17724992, rs1928295, rs2033529, rs2060604, rs2112347, rs2176598, rs2183825, rs2365389, rs2820292, rs2836754, rs2890652, rs3736485, rs3800229, rs3817334, rs3849570, rs3888190, rs4740619, rs4889606, rs543874, rs6091540, rs6457796, rs6477694, rs6567160, rs657452, rs6713510, rs6804842, rs7138803, rs7144011, rs7531118, rs7599312, rs7715256, rs7903146, rs879620, rs891389, rs9304665, rs9374842, rs943005, rs9540493, rs9579083, rs977747, rs9926784) from the analysis. Each red point depicts the IVW estimate using all SNPs. No single SNP is strongly driving the overall effect of BMI on colon polyps in this leave-one-out sensitivity analysis. BMI = body mass index, IVW = inverse variance weighting, SNPs = single-nucleotide polymorphisms.

**Figure 4.**
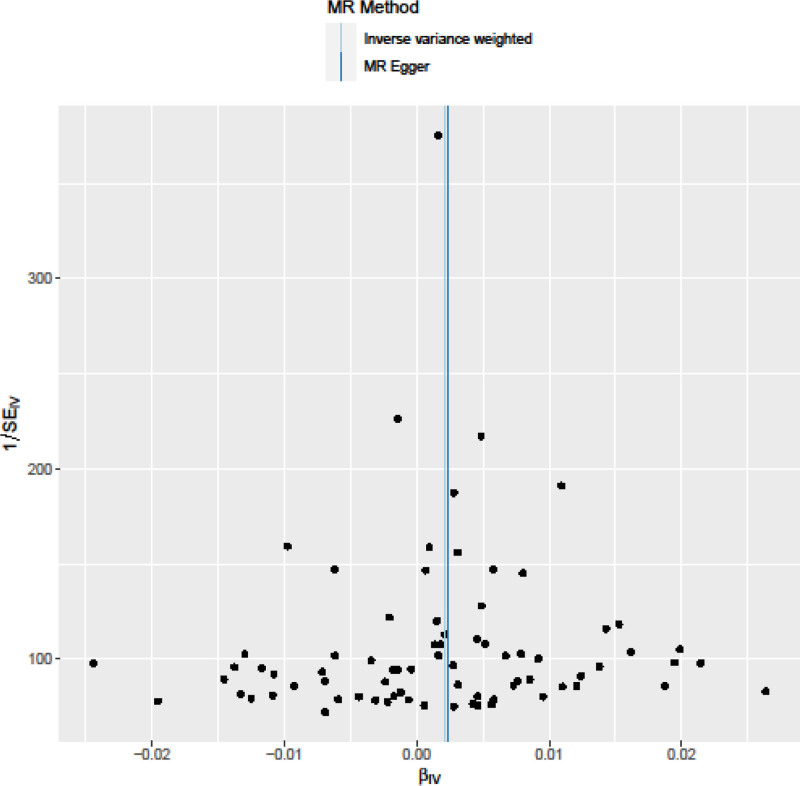
Funnel plot to assess heterogeneity. The blue line represents the inverse‐variance weighted estimate, and the dark blue line represents the Mendelian randomization‐Egger estimate.

Cochran Q test indicated no evidence of heterogeneity between IV estimates based on the individual variants (Table 2). Heterogeneity is the variability in the causal estimates obtained for each SNP (i.e., how consistent is the causal estimate across all SNPs). Low heterogeneity suggests increased reliability of MR estimates. Our results of *I*^2^ values showed low heterogeneity, indicating increased reliability of MR estimates (Table 2). Results from the “leave‐one‐out” analysis demonstrated that no single SNP was driving the IVW point estimate (Fig. 3). Asymmetry in the funnel plot indicates directional horizontal pleiotropy, which can bias MR methods; however, the funnel plot and MR Egger regression test showed no evidence of asymmetry (Fig. 4).

## 4. Discussion

Based on the GWAS summary data and employing SNPs that were substantially connected with BMI as instrumental factors, this study investigated the causal link between BMI and colon polyps using the two-sample MR approach. The findings indicated that BMI was a risk factor for colon polyps; the results of the random effects model IVW demonstrated that the risk of colon polyps increased 1.002 times for every unit increase in BMI; it is recommended that colon polyps prevention efforts focus more on the management of weight and BMI through dietary and lifestyle changes.

According to prior studies, body obesity as measured by BMI and the risk of colon polyps were both evaluated. In one cross-sectional investigation, Lai discovered that patients with a BMI ≥ 27 had a higher probability of developing rectosigmoid hyperplastic polyps than those with a BMI < 27 (odds ratio = 1.59, 95% confidence interval = 1.10–2.28, *P* < .05).^[13]^ Additionally, an observational study also demonstrate obesity were associated with a higher risk of digestive polyps in China.^[14]^ The relationship between BMI and colon polyps is still controversial. An earlier study of the Korean population found that, obesity is not associated with colonic adenomatous polyp in Korean population.^[5]^ Currently most of these studies are observational studies based on Asian populations.^[15,16]^

Colonic polyps may develop as a result of obesity via a number of different pathways. The mechanism may involve the increased secretion of inflammatory substances by the fat in obese patients, which results in an inflammatory state in the body and contributes to the activation of the transcriptional pathway that leads to colorectal cancer.^[17]^ Additionally, obese people frequently have insulin resistance, which results in hyperinsulinemia and promotes carcinogenesis and hyperplasia of the colon epithelium.^[18]^ A study from Japan showed that obesity can change the intestinal mucosa associated microbiota, which may be related to the development of colorectal tumors.^[19]^

The BMI predicted by gene prediction was not connected to colorectal precancerous lesions, according to a Mendelian randomization study. Given the large confidence interval, which includes the measured BMI, it is possible that weak power led to inaccurate results.^[20]^ The BMI of patients also has important guiding significance for the daily diagnosis and treatment of colon surgery. A study showed that there was still a negative correlation between BMI and CIT at different learning stages of endoscopists, suggesting that BMI should be a predictor of cecal intubation difficulties during colonoscopy.^[21]^

Our study is the first to use a large- population sample to investigate the causal link between obesity and colon polyps. According to our perspective, obese people should routinely get surveillance colonoscopies to find colorectal abnormalities early, such as adenomas and hyperplastic polyps, due to the danger of colon polyps developing into cancer.

However, the findings of this study need to be confirmed in Asian populations, along with clinical and randomized controlled trials, due to the dearth of GWAS data in Asian populations. Additionally, colon polyps were used in this investigation as the final result. Due to the incomplete data, the study was unable to establish a causal determination on high-risk polyps such as serrated polyps because it did not include the pathological information of colon polyps. There are many classification methods for colon polyps, such as NICE and PIT pattern. The risk of different types of polyps evolving into colon cancer is also different. At present, there is a lack of GWAS data of high-risk polyps, which makes the BMI-colon polyps-colon cancer pathway lack of more convincing data confirmation. Future prospective research will be required to investigate obesity’s connection to high-risk polyps.^[22]^

## 5. Conclusion

In summary, the results of MR analysis support that BMI may be causally associated with an increased risk of colon polyps. Our findings suggest that obesity may play an important role in the development of colon polyps. The current findings may provide an opportunity to determine the mechanisms underlying the effects of obesity on the risk of colon polyps.

## Acknowledgments

This study was supported by Guangdong Provincial Key Laboratory of Digestive Cancer Research (No. 2021B1212040006) and Sanming Project of Medicine in Shenzhen (No. SZSM201911010), Shenzhen Key Medical Discipline Construction Fund (No. SZXK016), Clinical research fund of the seventh Affiliated Hospital of Sun Yat sen University (ZSQYLCKYJJ202021).

## Author contributions

**Conceptualization:** Hui-Long Guo, Xin-Jie Ning, Jian-Feng Li, Jing-Yao Chen.

**Data curation:** Hui-Long Guo, Xin-Jie Ning, Jian-Feng Li, Jing-Yao Chen.

**Formal analysis:** Hui-Long Guo, Xin-Jie Ning, Jian-Feng Li, Jing-Yao Chen.

**Investigation:** Hui-Long Guo, Xin-Jie Ning, Jian-Feng Li, Jing-Yao Chen.

**Methodology:** Hui-Long Guo, Xin-Jie Ning, Jian-Feng Li, Jing-Yao Chen.

**Project administration:** Hui-Long Guo, Xin-Jie Ning, Jian-Feng Li, Jing-Yao Chen.

**Resources:** Hui-Long Guo, Xin-Jie Ning, Jian-Feng Li, Jing-Yao Chen.

**Software:** Hui-Long Guo, Xin-Jie Ning, Jian-Feng Li, Jing-Yao Chen.

**Supervision:** Hui-Long Guo, Xin-Jie Ning, Jian-Feng Li, Jing-Yao Chen.

**Validation:** Hui-Long Guo, Xin-Jie Ning, Jian-Feng Li, Jing-Yao Chen.

**Visualization:** Hui-Long Guo, Xin-Jie Ning, Jian-Feng Li, Jing-Yao Chen.

**Writing – review & editing:** Hui-Long Guo, Jing-Yao Chen.

**Writing – original draft:** Xin-Jie Ning, Jian-Feng Li.

## Supplementary Material


