# Supplementary material for: Causal association between body mass index and risk of colon polyps: A Mendelian randomization study
Source: Medicine (Baltimore). 2025 May 23;104(21):e42022. doi: 10.1097/MD.0000000000042022 (PMC12113971; doi:10.1097/MD.0000000000042022)
Supplement: Supplementary file 1 [file medi-104-e42022-s001.pdf]

Table1. Information of 76 SNPs significantly related to BMI

SNP	EAF	beta	SE	R^2	F
rs1000940	0.225	0.0184	0.0033	6.71411E-05	31.09
rs10132280	0.3333	-0.0221	0.0033	9.68555E-05	44.85
rs1016287	0.675	-0.0228	0.0033	0.000103088	47.74
rs10182181	0.5	0.0309	0.0029	0.000245146	113.53
rs10733682	0.575	-0.0188	0.003	8.48098E-05	39.27
rs10840100	0.725	0.0206	0.003	0.000101826	47.15
rs11030104	0.2	-0.0416	0.0037	0.000272944	126.41
rs11057405	0.0917	-0.0304	0.0053	7.10517E-05	32.90
rs11165643	0.575	0.0221	0.003	0.000117193	54.27
rs11672660	0.175	-0.0339	0.0038	0.000171857	79.58
rs1167827	0.5417	0.02	0.0031	8.98891E-05	41.62
rs11727676	0.075	-0.0365	0.0063	7.24908E-05	33.57
rs12286929	0.4333	0.0211	0.0029	0.000114322	52.94
rs12429545	0.1	0.0324	0.0044	0.000117096	54.22
rs12448257	0.225	0.0246	0.0037	9.5463E-05	44.20
rs12940622	0.4583	-0.0183	0.0029	8.5996E-05	39.82
rs12986742	0.5	0.0207	0.0036	7.14026E-05	33.06
rs13021737	0.875	0.0604	0.0039	0.000517761	239.85
rs13078960	0.1833	0.029	0.0038	0.000125772	58.24
rs13107325	0.1167	0.0472	0.0066	0.000110448	51.14
rs13130484	0.4333	0.0398	0.003	0.000379987	176.00
rs13191362	0.2	-0.0285	0.0047	7.94089E-05	36.77
rs13201877	0.0833	0.0236	0.0043	6.50532E-05	30.12
rs13329567	0.2167	-0.0307	0.0035	0.000166142	76.94
rs1421085	0.45	0.0803	0.003	0.001544994	716.45
rs1441264	0.55	0.0172	0.0031	6.64836E-05	30.78
rs1460676	0.2167	0.0209	0.0038	6.53291E-05	30.25
rs14810	0.675	0.0183	0.0033	6.64133E-05	30.75
rs1516725	0.9083	0.0448	0.0044	0.000223853	103.67
rs1528435	0.5833	0.0175	0.003	7.34871E-05	34.03
rs17001654	0.1583	0.0304	0.0052	7.38105E-05	34.18
rs17066856	0.1333	-0.0371	0.005	0.000118896	55.06
rs17094222	0.2083	0.0249	0.0037	9.78053E-05	45.29
rs17203016	0.2	0.0211	0.0038	6.65853E-05	30.83
rs17381664	0.425	0.0201	0.0031	9.07902E-05	42.04
rs17724992	0.3083	-0.0196	0.0034	7.17683E-05	33.23
rs1928295	0.425	-0.0182	0.0029	8.50588E-05	39.39
rs2033529	0.2583	0.0183	0.0032	7.06287E-05	32.70
rs2060604	0.4417	-0.0203	0.003	9.88818E-05	45.79
rs2112347	0.375	-0.0254	0.003	0.000154799	71.68
rs2176598	0.8	-0.0185	0.0033	6.78728E-05	31.43

rs2183825	0.2917	0.0241	0.0032	0.000122487	56.72
rs2365389	0.3417	-0.0195	0.003	9.12424E-05	42.25
rs2820292	0.5083	0.0181	0.0029	8.41268E-05	38.95
rs2836754	0.65	0.0169	0.003	6.85347E-05	31.73
rs2890652	0.125	0.0279	0.0049	7.00157E-05	32.42
rs3736485	0.575	-0.016	0.0029	6.57393E-05	30.44
rs3800229	0.6917	0.0175	0.0032	6.45889E-05	29.91
rs3817334	0.45	0.0256	0.003	0.000157246	72.82
rs3849570	0.3667	0.0183	0.0033	6.64133E-05	30.75
rs3888190	0.3583	0.0311	0.003	0.000232053	107.47
rs4740619	0.4667	-0.017	0.0029	7.42129E-05	34.36
rs4889606	0.3583	-0.0187	0.003	8.391E-05	38.85
rs543874	0.2667	0.0497	0.0037	0.000389538	180.43
rs6091540	0.275	-0.0185	0.0033	6.78728E-05	31.43
rs6457796	0.2583	0.0209	0.0033	8.66237E-05	40.11
rs6477694	0.6417	-0.0169	0.003	6.85347E-05	31.73
rs6567160	0.2833	0.0562	0.0035	0.00055655	257.83
rs657452	0.5833	-0.0227	0.0031	0.000115794	53.62
rs6713510	0.4833	0.0164	0.0029	6.90671E-05	31.98
rs6804842	0.575	0.0183	0.003	8.0359E-05	37.21
rs7138803	0.4417	0.032	0.003	0.000245675	113.78
rs7144011	0.275	0.0274	0.0035	0.000132348	61.29
rs7531118	0.6083	0.0331	0.003	0.000262851	121.73
rs7599312	0.2917	-0.0214	0.0033	9.08176E-05	42.05
rs7715256	0.55	-0.0168	0.0029	7.24771E-05	33.56
rs7903146	0.25	-0.0235	0.0033	0.000109514	50.71
rs879620	0.5917	0.0244	0.0039	8.45324E-05	39.14
rs891389	0.325	0.0209	0.0037	6.89079E-05	31.91
rs9304665	0.7	0.0243	0.0043	6.89692E-05	31.94
rs9374842	0.7417	0.0196	0.0034	7.17683E-05	33.23
rs943005	0.1	0.0444	0.0038	0.000294768	136.52
rs9540493	0.55	-0.0182	0.0031	7.44383E-05	34.47
rs9579083	0.2333	0.0295	0.0046	8.88177E-05	41.13
rs977747	0.5333	-0.0168	0.003	6.77261E-05	31.36
rs9926784	0.2083	-0.0249	0.0038	9.27259E-05	42.94
